# One-Step Synthesis of High Pure Tris(8-hydroxyquinoline)aluminum for Optics and Photonics

**DOI:** 10.3390/ma15030734

**Published:** 2022-01-19

**Authors:** Roman Avetisov, Ksenya Kazmina, Artem Barkanov, Marina Zykova, Andrew Khomyakov, Alexander Pytchenko, Igor Avetissov

**Affiliations:** Department of Chemistry and Technology of Crystals, Mendeleev University of Chemical Technology, 125047 Moscow, Russia; armoled@mail.ru (R.A.); kazmina.k.v@muctr.ru (K.K.); barkanov.a.d@muctr.ru (A.B.); zykova_mp@inbox.ru (M.Z.); homa-ifh@yandex.ru (A.K.); pytchenkoaa@mail.ru (A.P.)

**Keywords:** tris(8-hydroxyquinoline)aluminum, pure substance, inductively coupled plasma mass spectrometry

## Abstract

A simple method of synthesis of high pure tris(8-hydroxyquinoline)aluminum (Alq_3_) from commercial available 5N Al_2_O_3_ and 8-hydroxyquinolinol has been developed. One-step exchange chemical reaction has been conducted under controlled 8-hydrixyquinoline vapor at a temperature of 190–240 °C with water removal by phosphorus anhydride. According to analysis of inductively coupled plasma mass-spectrometry, the chemical purity of synthesized Alq_3_ was 99.998 wt%. Photoluminescence of the synthesized Alq_3_ has been measured and slightly differed from those of Alq_3_ obtained by traditional organic synthesis.

## 1. Introduction

In the last two decades, a large effort has been directed towards development of wearable/implantable electronics based on organic semiconducting materials. The most recent developments include environmental monitoring, implantable medical devices, on-skin sensors, and disposable plastic electronics such as e-tickets, RFID tags, plastic cards, etc. [1,2,3,4,5,6,7,8,9,10,11,12,13,14]. These devices require plentiful and low-cost materials and production technologies supporting their dynamic development. Organic semiconducting material technologies are interesting for their fine-tuning of characteristics due to the large variability of chemical formulas at high productivity and low production costs.

To date OLED technologies have become widespread in various fields of techniques: perfect TV displays, energy efficient lighting devices, IR sensors and displays for medical application, etc. Tris(8-hydroxyquinoline)aluminum (Alq_3_) was the first OLED emission material [15] and till now it has a wide application both as an emitting material and an electron transport material for cheap commercial devices [1].

**Figure 1:**
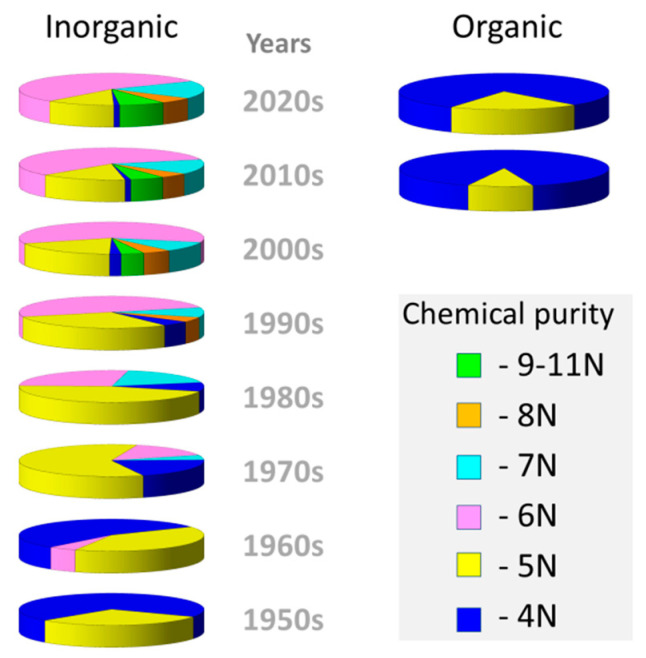
Production dynamic (rel.%) of inorganic (left column) and organic (right column) semiconducting materials having different chemical purity (xN).

Conceptually, OLED devices are electronic semiconductor structures [6], and, as in the case of inorganic semiconductors, organic materials that are used in multilayer OLED structures must meet the requirements for semiconductors. In particular, the chemical purity of organic semiconductors must be as high as inorganic ones. The successful development of technologies for inorganic semiconductors and devices based on them began more than 70 years ago, just when the chemical purity of 99.999 wt% (5N) became generally available (Figure 1). To date, modern inorganic semiconductors are characterized by a purity of 99.99999 wt% (7N) for GaAs technologies [16] to 99.999999999 wt% (11N) for silicon technologies [17].

Organic semiconducting materials (including phosphors for OLED technology) are generally produced by conducting of a chemical reaction in a complex liquid media. This technology needs pure solvents, organic precursors and multistep purification procedures.

For instance, the production of high pure Alq_3_ (99.995 wt%) still remains a comparatively expensive: the price for sublimated Alq_3_ 99.9–99.999 wt% is 60–100 kEuro/kg [18,19]. The standard procedure includes the reaction of complex formation of aluminum salt (chloride, nitrate) with 8-hydroxyquinolinol in isopropanol solution with further sedimentation by ammonia hydroxide, multiply washing by isopropanol, and finally vacuum sublimation [20]. This procedure needs high pure initial reagents and special extra-pure chemical equipment.

In the presented research, we developed a simple one-step method of synthesis of tris(8-hydroxyquinolinate)aluminum from cheap commercial preparations with 5N and 6N chemical purity. The synthesized Alq_3_ preparation was formed on the surface of Al_2_O_3_ grains and the synthesized heterophase preparation could be a source of the chemical pure Alq_3_ for OLED technology.

## 2. Materials and Methods

### 2.1. Impurity Determination by ICP-MS

To analyze chemical purity of initial and final preparations, we used inductively coupled plasma mass spectrometry with preliminary transfer of the solid sample to the liquid phase by dissolving them in high-purity nitric (HNO_3_) acid (7N7), purified by a Berghof BSB-939-IR surface distillation system (Berghof GmbH, Eningen, Germany) or high-purity sulfuric (H_2_SO_4_) acid (8N Ultrapur, Sigma-Aldrich Chemie GmbH, Taufkirchen, Germany) in a SPEEDWAVE-FOUR microwave decomposition system (Berghof GmbH, Eningen, Germany) equipped with DAP-100 PTFE autoclaves (Berghof GmbH, Eningen, Germany). We used extra pure water (AquaMax-Ultra 370 Series, Young Lin Instruments Co., Ltd., Anyang, South Korea) with a specific resistance of 18 MΩ·cm for dilution.

Analytical measurements were carried out on a NexION 300D inductively coupled plasma mass spectrometer (ICP-MS) (PerkinElmer Inc., Waltham, MA, USA). The TotalQuant method for determination of 65 chemical elements’ concentrations was used [21] with the operating parameters presented in Table 1.

### 2.2. Initial Preparations

**Figure 2:**
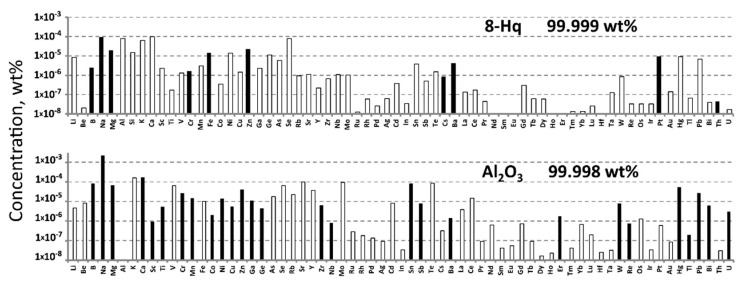
Impurity concentrations determined by ICP-MS in the initial preparations. Here and after, the empty (white) bars indicate the limits of determination (LD) of ICP-MS analysis. The concentrations of the non-presented elements were less 10^−8^ wt%.

Powder Al_2_O_3_ purchased from Prima Ltd. (Korolev, Russia) was used as an Al-source for Alq_3_ synthesis. The above preparation is usually used for sapphire crystal growth for laser applications. According to the ICP-MS analysis (Figure 2), it was as pure as 99.998 wt% (65 elements detected). A preliminary heat-treated (870 K) Al_2_O_3_ powder preparation was used in a synthesis procedure.

8-hydroxyquionolinol (8-Hq) purchased from Komponent Reaktive Ltd. (Moscow, Russia) was additionally purified by vacuum sublimation to the chemical purity of 99.999 wt% determined by 65 elements (Figure 2). As-sublimated 8-Hq was used directly in a synthesis procedure.

### 2.3. SEM and Optical Microscopy Analysis

To analyze the preparation morphology, we used optical and electron microscopies. We used a Stereo Discovery V.12 binocular microscope (CarlZeiss, Oberkochen, Germany) with white and UV lighting.

SEM images of powder preparations were obtained using a VEGA-3 LMU scanning electron microscope (TESCAN ORSAY HOLDING, Brno–Kohoutovice, Czech Republic) in secondary electron (SE) mode with 5 kV accelerating voltage.

### 2.4. Spectral Parameter Measurements

All of the luminescence measurements were carried out at room temperature. We used a Fluorolog FL3-22 spectrofluorimeter (Horiba Jobin Yvon, Longjumeau, France) with double-grating excitation and emission monochromators for luminescence measurements over 400 to 700 nm wavelength range with a 0.1 nm step. PL spectra deconvolutions were carried out with OriginPro 8 SR4 (OriginLab Corp., Northampton, MA, USA) software using the Fit Multiple Peak procedure. The luminescence decay kinetics were studied by the excitation of a pulsed diode laser (λ = 377 nm, Δτ = 1.5 ns) and a Xenon 450W Ushio UXL-450S/O lamp (355 nm). Processing of the luminescence decay curves was carried out using the Fit Exponential procedure of an OriginPro 8 SR4 software. All of the decay curves were described by two exponentials (criterion Adj. R-Square > 0.998). The final data were averaged over 5 measurements.

## 3. Results and Discussion

The general idea of high pure substances synthesis is that the best results could be obtained when we used the minimal set of initial preparations to conduct the synthesis reaction.

In our case the formal synthesis was described by the heterophase reaction (1)

(1) $${\mathrm{Al}}_{2}O_{3}^{s}+3\;8{\mathrm{Hq}}^{v}\to {\;\mathrm{Alq}}_{3}^{s}+3{\;H}_{2}O^{v}$$

**Figure 3:**
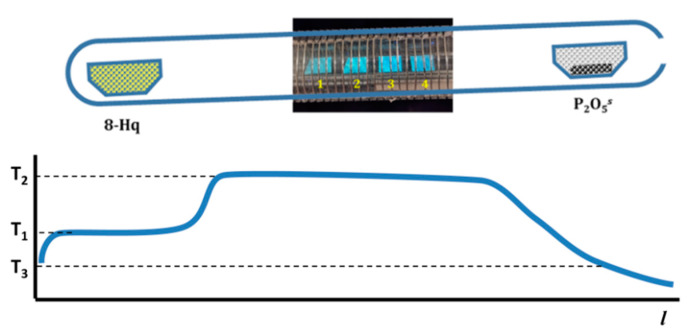
Scheme of setup for synthesis of Alq_3_ and temperature distribution in the setup.

The heterophase synthesis was conducted in a two-zone resistive furnace in a quartz-glass reactor (Figure 3). The 8-Hq source was placed at 328–333 K (T_1_) at the closed end of the reactor, while Al_2_O_3_ powder was placed in a hot zone at 463–513 K (T_2_). To move the equilibrium towards the reaction products, we captured the water vapor by solid preliminary dried P_2_O_5_, which was placed in the quartz glass vessel at the cold open end of the reactor at temperature about 308–318 K (T_3_). There was no need to put a pre-desiccant between P_2_O_5_, and the open end of the tube with its total length 20 cm because the flux of 8-Hq and H_2_O vapors was directed from the closed end to the open end of the reactor. During the test experiments without Al_2_O_3_ preparation, we did not observe P_2_O_5_ degradation for 50 h of the processing.

**Figure 4:**
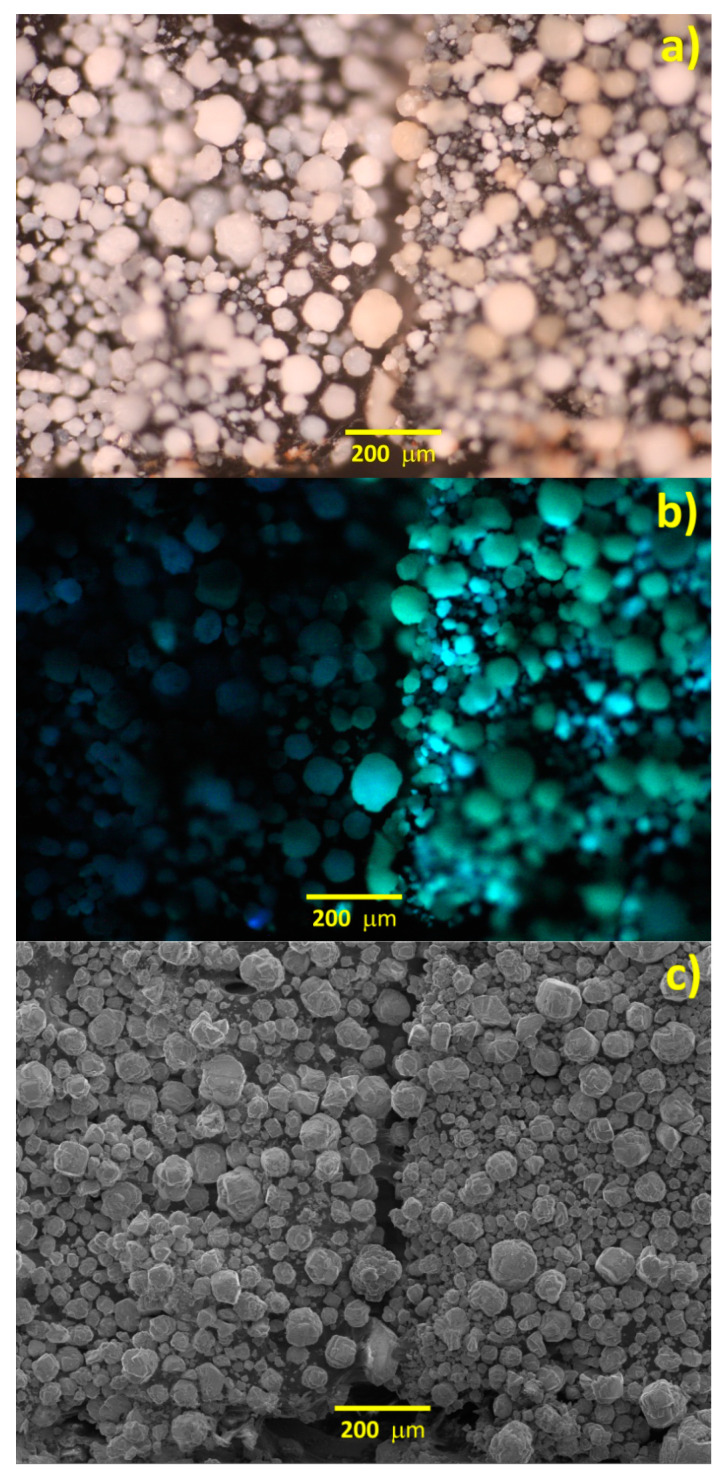
Microphotographs (**a**,**b**) and SEM image (**c**) of powder Al_2_O_3_ preparations under day light (**a**) and UV lighting (**b**) before (left half) and after heat treatment under 8-Hq vapor (right half).

Analysis of grains morphology after synthesis showed that the grain size distribution was the same as for the initial Al_2_O_3_ powder (Figure 4a). However, under UV lighting, we observed bright green—yellowish photoluminescence for the grains treated under 8-Hq vapor (Figure 4b right half). SEM analysis in SE mode showed that the number of output secondary electrons form initial Al_2_O_3_ grains (Figure 4c left half) was more than that from the grains treated under 8-Hq vapor (Figure 4c right half), because we observed a brighter image for the initial Al_2_O_3_ grains. All these observations indicated that we synthesized a new compound on Al_2_O_3_ grains surface and the thickness of the product was very small.

**Figure 5:**
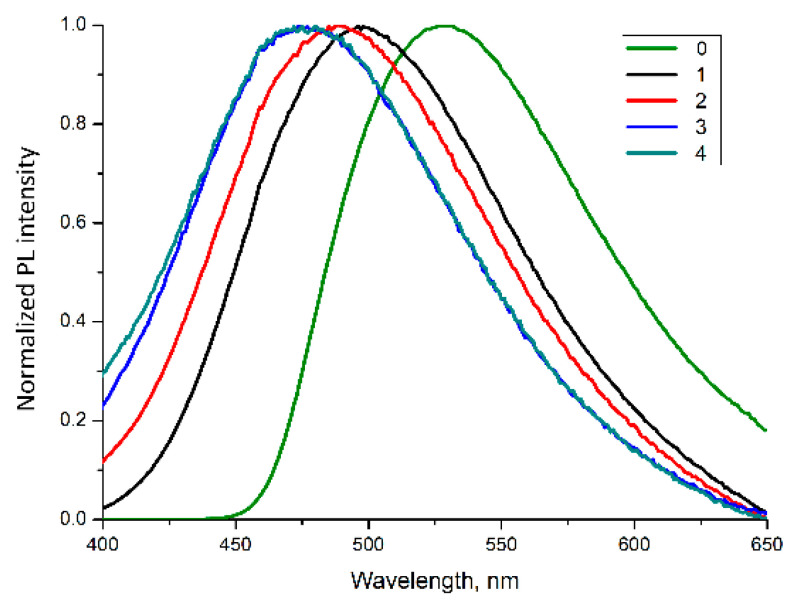
Normalized PL spectra (λ^exc^ = 365 nm) of Alq_3_ samples synthesized by the direct reaction at T = 463 K and by wet synthesis. The numbers correspond to the position of samples in the furnace at the high-temperature synthesis (see Figure 1 and Table 2).

Spectral analysis showed that depending on a sample position in the furnace (see Figure 3) the $\lambda_{\mathrm{PL}}^{\max}$ shifted from 496 nm to 474 nm ($\lambda^{\mathrm{exc}}$ = 365 nm) (Figure 5) with the corresponding increase of PL intensity more than in seven times (Table 2).

Low PL intensity of direct-synthesized Alq_3_ preparations comparing to the wet-synthesized Alq_3_ we explained by very small thickness of the synthesized compound on the surface of Al_2_O_3_ grains. The hypsochromic shift of PL maximum for the direct-synthesized Alq_3_ comparing with the wet-synthesized Alq_3_ could results from summarizing of PL lighting with the reflected excitation light from the interface surface of Alq_3_ and Al_2_O_3_. We must also take into consideration the scheme of polymorph transformation for Alq_3_ [22]. In the case of δ-Alq_3_ (or γ-Alq_3_) the PL maximum in the films was found to be 474 nm [23].

Analysis of PL decay kinetics (Table 3, Figures S1–S5) showed that they were successfully described by two-exponential equation. The short-lived centers had the lifetime about 2 ns, while the long-lived centers had the lifetime of 16–17 ns. We observed that the wet–synthesized sample has a decay kinetics specific for α-Alq_3_ [20], and it was longer than that for the samples obtained by the direct synthesis.

We failed to find any data on PL decay kinetics for different polymorphs of Alq_3_ in the literature. Therefore, we assumed that for δ-Alq_3_ (or γ-Alq_3_), which were probably obtained in our experiments, the PL decay kinetics was shorter than for α-Alq_3_.

**Figure 6:**
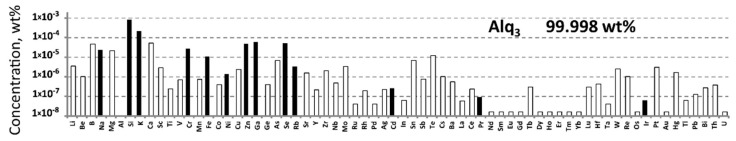
Impurity concentrations determined by ICP-MS in as-synthesized Alq_3_.

According to ICP-MS analysis, as-synthesized Alq_3_ has the chemical purity of 99.998 wt% (Figure 6). The major impurities were Si and K. We assume these impurities are inherited from the container material: a quartz-glass reactor and a quartz-glass vessel with 8-Hq.

High purity aluminum oxide is often termed as high purity alumina (HPA). It is a high-value, white, granular chemical produced commercially. Analysis of the world alumina market showed that 5N and 6N Al_2_O_3_ are available preparation at a comparatively low price [24]. 8-Hq is simply purified by a sublimation procedure to the level of 99.999 wt%. Thus, we could say that there are commercially available sources for simple synthesis of high pure Alq_3_.

## 4. Conclusions

A new approach to the synthesis of tris(8-hydroxyquinolate) aluminum showed the fundamental possibility of preparation an electroluminescent high-purity material using fairly simple operations and an easy procedure. One of the advantages of the produced material is its stability to the environment. We did not observe any degradation when storage the synthesized preparations in common used vessels without additional sealing or filling with an inert gas. Further refinement of the developed technique, for instance, using glassy carbon reactor, will make it possible to obtain cheap and even more high-pure materials for OLED technologies.

## Figures and Tables

**Table 1:** The operating mode of the NexION 300D instrument for conducting impurity analysis of samples.

Nebulizer type	Concentric (Meinhard), PFA
Spray chamber	Scott double-pass chamber, PFA
Argon flow rate, L/min	
through the nebulizer	0.96
plasma-forming	15
auxiliary	1.2
Generator power, W	1450
Collision gas (He) flow rate, L/min	4.6
Number of scan cycles	8

**Table 2:** PL peaks parameters for Alq_3_ samples synthesized by the direct synthesis and by the wet technique [20] (number 0).

Number	Peak Area	FWHM, nm	Center, nm	Height, cps
0	1.05 × 10^10^	113.00	527	8.69 × 10^7^
1	8.43 × 10^8^	113.94	496	7.04 × 10^6^
2	3.40 × 10^8^	118.47	489	2.72 × 10^6^
3	2.03 × 10^8^	119.47	480	1.61 × 10^6^
4	1.31 × 10^8^	122.47	474	1.02 × 10^6^

**Table 3:** PL decay kinetics of Alq_3_ samples synthesized by the direct synthesis and by the wet technique [20] (number 0), described by the equation Y = A1 × exp(−x/τ1) + A2 × exp(−x/τ2) + Y0.

Number	$\lambda_{PL}^{max},\;$ nm	Y0	A1	τ1, ns	A2	τ2, ns
0	527	102.39 ± 0.71	6503 ± 134	8.56 ± 0.19	8945 ± 172	21.14 ± 0.15
1	496	127.54 ± 1.01	41694 ± 822	2.78 ± 0.03	5694 ± 58	16.91 ± 0.11
2	489	80.30 ± 0.92	39579 ± 800	2.71 ± 0.03	6413 ± 50	17.23 ± 0.09
3	480	45.02 ± 0.87	51000 ± 1319	2.31 ± 0.03	6596 ± 44	16.67 ± 0.08
4	474	30.73 ± 0.86	78664 ± 2359	1.97 ± 0.02	6395 ± 40	16.17 ± 0.07

## Supplementary Materials

The following supporting information can be downloaded at: https://www.mdpi.com/article/10.3390/ma15030734/s1, Figure S1: PL decay kinetics at 527 nm for the Alq3 preparation obtained by the «wet» synthesis, Figure S2: PL decay kinetics at 496 nm for the Alq3 preparation (N1) obtained by the direct synthesis, Figure S3: PL decay kinetics at 489 nm for the Alq3 preparation (N2) obtained by the direct synthesis, Figure S4: PL decay kinetics at 480 nm for the Alq3 preparation (N3) obtained by the direct synthesis, Figure S5: PL decay kinetics at 474 nm for the Alq3 preparation (N4) obtained by the direct synthesis.

## References

[1] So F. (2010). Organic Electronics: Materials, Processing, Devices and Applications.

[2] Irimia-Vladu M., Glowacki E.D., Sariciftci N.S., Bauer S. (2018). Green Materials for Electronics.

[3] Wu X., Ma Y., Zhang G., Chu Y., Du J., Zhang Y., Li Z., Duan Y., Fan Z., Huang J. (2015). Thermally Stable, Biocompatible, and Flexible Organic Field-Effect Transistors and Their Application in Temperature Sensing Arrays for Artificial Skin. Adv. Funct. Mater..

[4] Stadlober B., Zirkl M., Irimia-Vladu M. (2019). Route towards Sustainable Smart Sensors: Ferroelectric Polyvinylidene Fluoride-Based Materials and Their Integration in Flexible Electronics. Chem. Soc. Rev..

[5] Nalwa H.S. (2008). Handbook of Organic Electronics and Photonics.

[6] Anthony J.E., Arias A.C., Bergsmann M., Blanchet G., Cantatore E., Halik M., Heuken M., Horowitz G., Huang J., Katz H.E., Klauk H. (2006). Organic Electronics.

[7] Peyghambarian N., Fallahi M., Piprek J., Sun S., Prigodin V.N., Epstein A.J., Meng X., Zhu W., Tian H., Li Y. (2016). Introduction to Organic Electronic and Optoelectronic Materials and Devices.

[8] Wong W.S., Salleo A. (2009). Flexible Electronics: Materials and Applications.

[9] Shinar R., Shinar J. (2009). Organic Electronics in Sensors and Biotechnology.

[10] Bao Z., Locklin J., Bao Z., Locklin J. (2018). Organic Field-Effect Transistors.

[11] Martins J., Sousa L. (2009). Bioelectronic Vision.

[12] Brabec C.J., Dyakonov V., Parisi J., Sariciftci N.S. (2013). Organic Photovoltaics: Concepts and Realization.

[13] Perlin J., Hepp A.F., Bailey S.G., Raffaelle R.P., Blankenship R.R., Lane P.A., Kafafi Z.H., Persson N., Inganas O., Gregg B.A., Sam-Shajing S., Niyazi Serdar S. (2017). Organic Photovoltaics.

[14] Brutting W. (2005). Physics of Organic Semiconductors.

[15] Tang C.W., VanSlyke S.A. (1987). Organic Electroluminescent Diodes. Appl. Phys. Lett..

[16] Electronic Grade Gallium Arsenide. https://chem.libretexts.org/@go/page/212888.

[17] Maurits J.E.A. (2014). Silicon Production. Treatise on Process Metallurgy.

[18] https://www.americanelements.com/tris-8-hydroxyquinoline-aluminum-2085-33-8.

[19] https://cymitquimica.com/products/TR-T875045/2085-33-8/tris8-hydroxychinolinaluminum/.

[20] Avetissov I.C., Akkuzina A.A., Avetisov R.I., Khomyakov A.V., Saifutyarov R.R. (2016). Non-Stoichiometry of Tris(8-Hydroxyquinoline) Aluminium: Is It Possible?. CrystEngComm.

[21] Pruszkowski E., Life P.E. (2004). Total quant analysis of teas and wines by ICP-MS. Perkin Elmer Life and Analytical Sciences.

[22] Avetisov R.I., Akkuzina A.A., Cherednichenko A.G., Khomyakov A.V., Avetissov I.C. (2014). Polymorphism of Tris(8-hydroxyquinoline)aluminum, Gallium, and Indium. Dokl. Chem..

[23] Muccini M., Loi M.A., Kenevey K., Zamboni R., Masciocchi N., Sironi A. (2004). Blue Luminescence of Facial Tris(Quinolin-8-Olato)Aluminum(III) in Solution, Crystals, and Thin Films. Adv. Mater..

[24] https://www.persistencemarketresearch.com/market-research/high-purity-alumina-market/toc.

