# Supplementary material for: One-Step Synthesis of High Pure Tris(8-hydroxyquinoline)aluminum for Optics and Photonics
Source: Materials (Basel). 2022 Jan 19;15(3):734. doi: 10.3390/ma15030734 (PMC8837004; doi:10.3390/ma15030734)
Supplement: Supplementary file 1 [file materials-15-00734-s001.zip › materials-1508286-supplementary.pdf]

materials

MDPI

Supplementary

One-step Synthesis of High Pure Tris(8-oxyquinoline)aluminum For Optics And Photonics

Roman Avetisov, Ksenya Kazmina, Artem Barkanov, Marina Zykova, Andrew Khomyakov, Alexander Pytchenko and Igor Avetissov

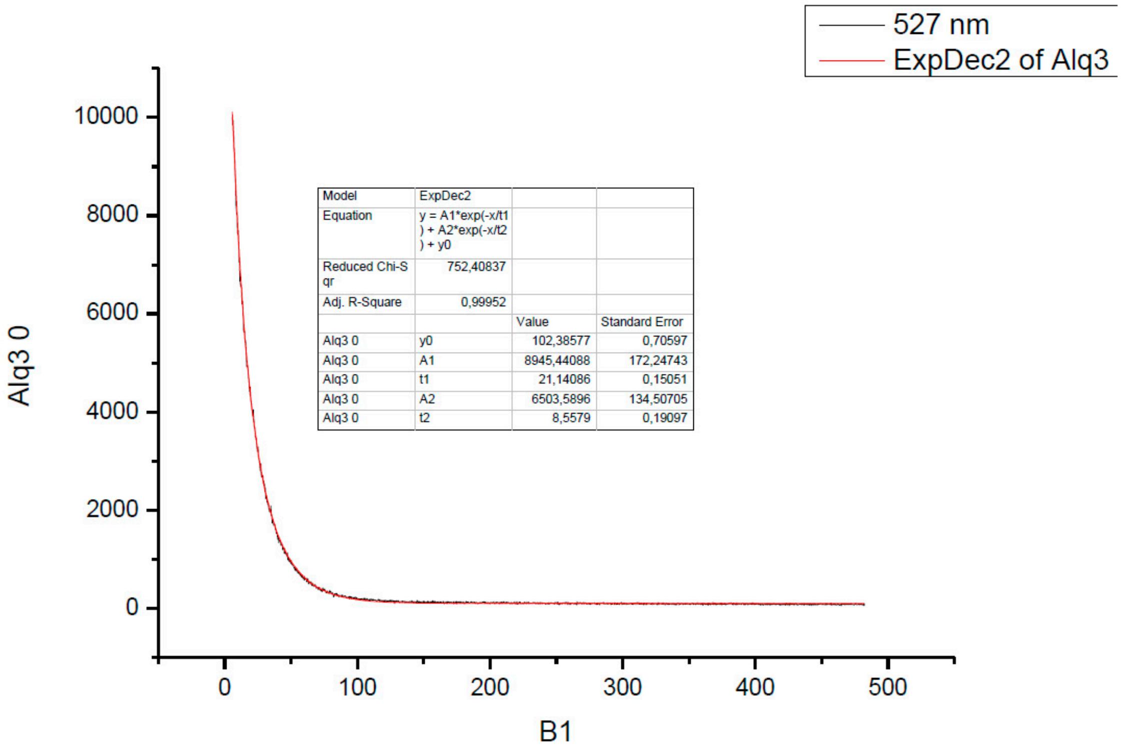

Figure S1. PL decay kinetics at 527 nm for the Alq3 preparation obtained by the «wet» synthesis as described in Ref. [Avetissov, I. C.; Akkuzina, A. A.; Avetisov, R. I.; Khomyakov, A. V.; Saifutyarov, R. R. Non-Stoichiometry of Tris(8- Hydroxyquinoline) Aluminium: Is It Possible? CrystEngComm 2016, 18 (12), 2182–2188. https://doi.org/10.1039/C6CE00011H.].

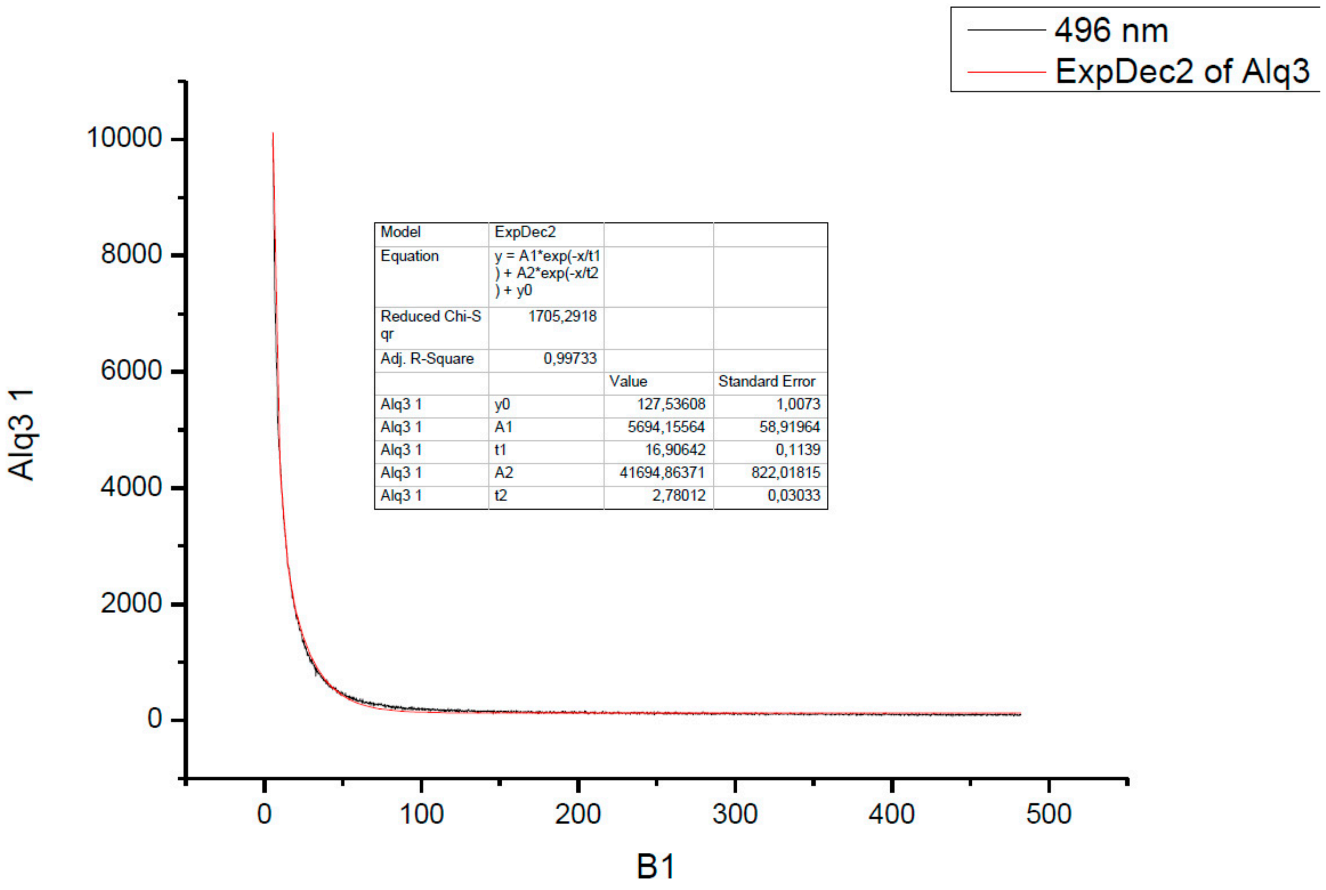

Figure S2. PL decay kinetics at 496 nm for the Alq3 preparation (N1) obtained by the direct synthesis.

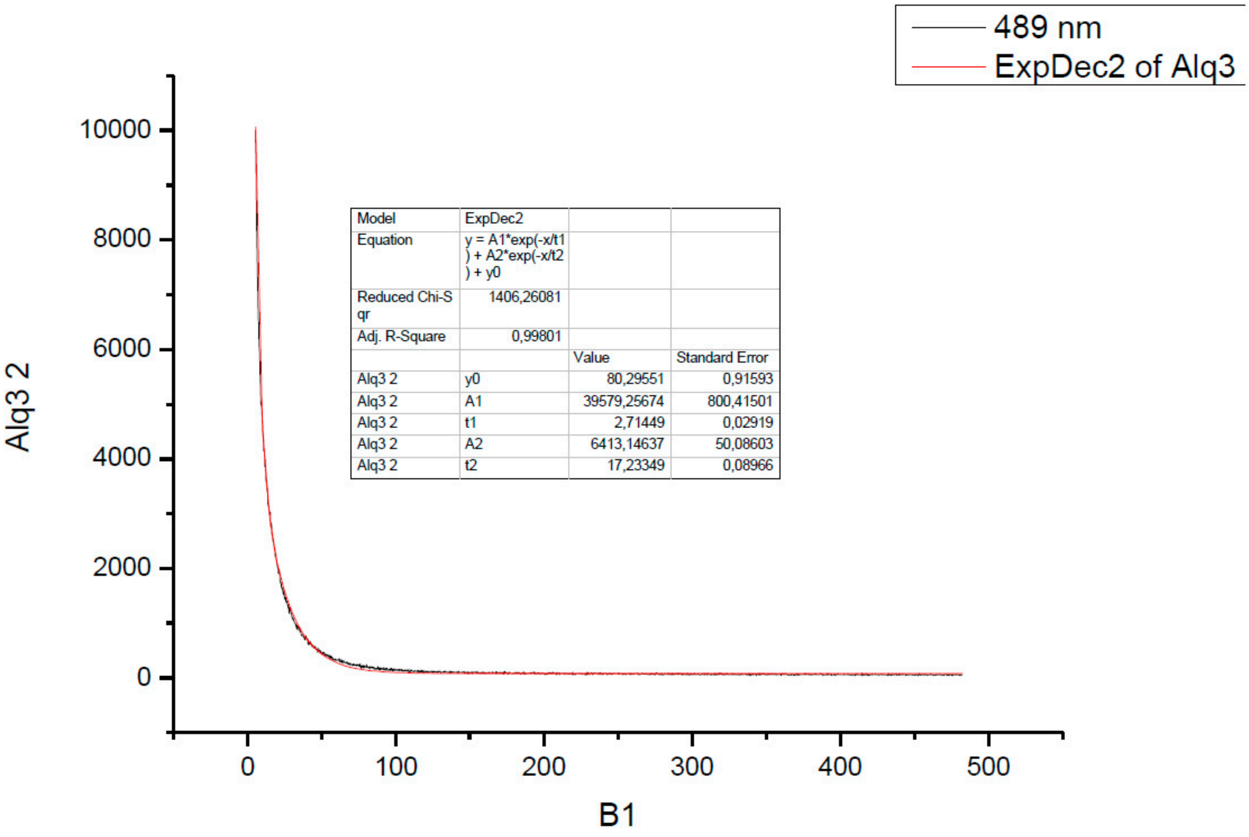

Figure S3. PL decay kinetics at 489 nm for the Alq3 preparation (N2) obtained by the direct synthesis.

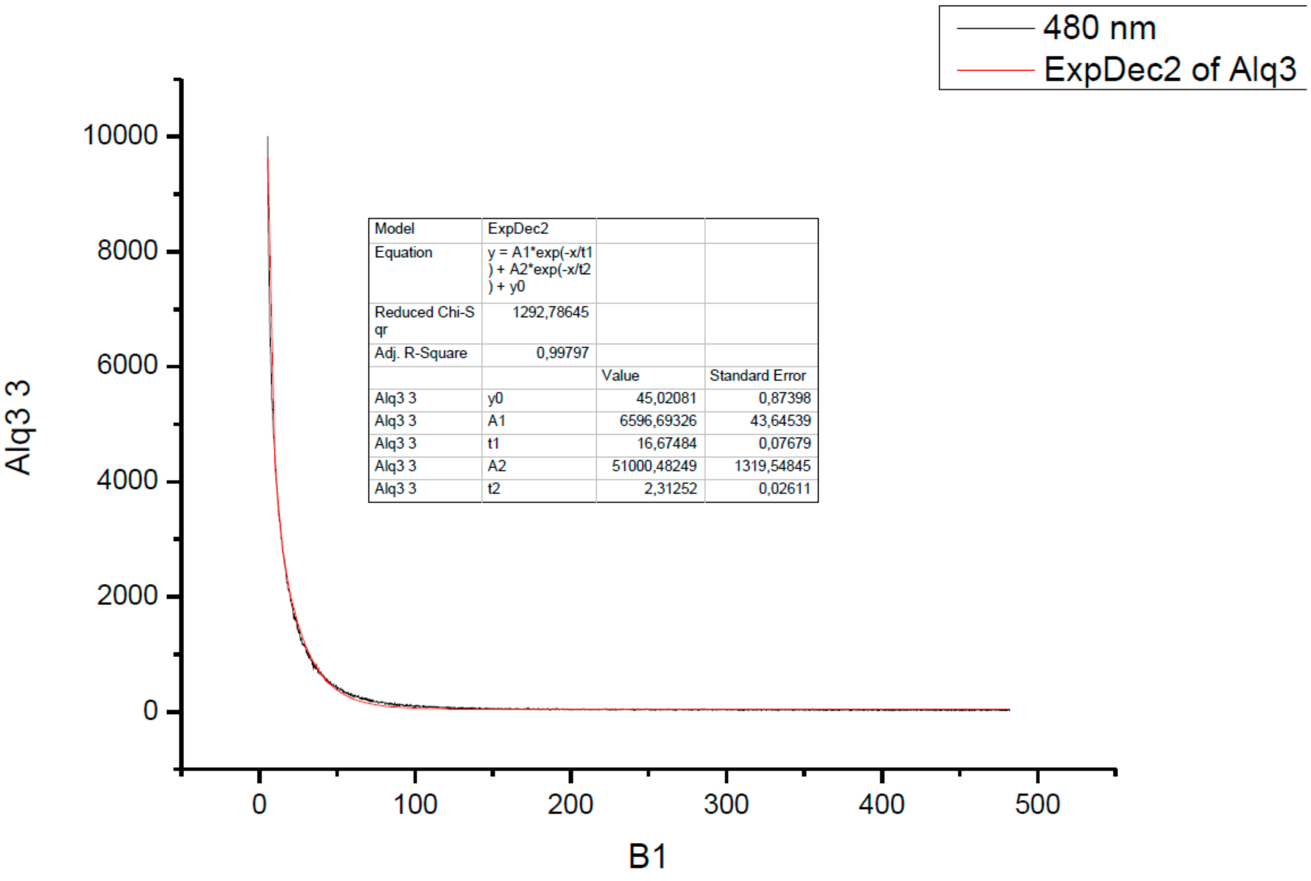

Figure S4. PL decay kinetics at 480 nm for the Alq3 preparation (N3) obtained by the direct synthesis.

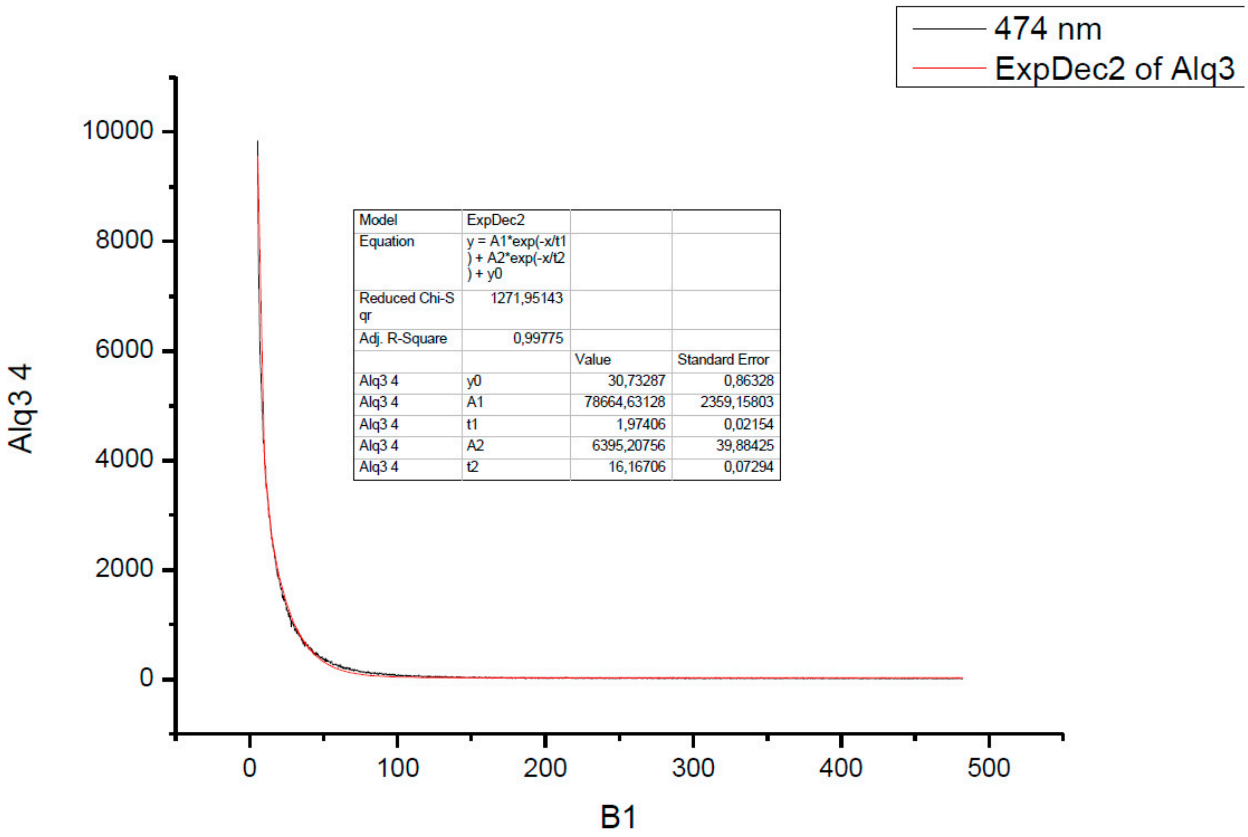

Model	ExpDec2		
Equation	y = A1*exp(-x/t1) + A2*exp(-x/t2) + y0		
Reduced Chi-Sqr	1271,95143		
Adj. R-Square	0,99775		
		Value	Standard Error
Alq3 4	y0	30,73287	0,86328
Alq3 4	A1	78664,63128	2359,15803
Alq3 4	t1	1,97406	0,02154
Alq3 4	A2	6395,20756	39,88425
Alq3 4	t2	16,16706	0,07294

Figure S5. PL decay kinetics at 474 nm for the Alq3 preparation (N4) obtained by the direct synthesis.
